# The Role of MSCs and Cell Fusion in Tissue Regeneration

**DOI:** 10.3390/ijms222010980

**Published:** 2021-10-12

**Authors:** Jessica Dörnen, Thomas Dittmar

**Affiliations:** Institute of Immunology, Center for Biomedical Education and Research (ZBAF), Witten/Herdecke University, Stockumer Str. 10, 58448 Witten, Germany; jessica.doernen@uni-wh.de

**Keywords:** tissue regeneration, cell fusion, mesenchymal stem/progenitor cells

## Abstract

Regenerative medicine is concerned with the investigation of therapeutic agents that can be used to promote the process of regeneration after injury or in different diseases. Mesenchymal stem/stromal cells (MSCs) and their secretome—including extracellular vesicles (EVs) are of great interest, due to their role in tissue regeneration, immunomodulatory capacity and low immunogenicity. So far, clinical studies are not very conclusive as they show conflicting efficacies regarding the use of MSCs. An additional process possibly involved in regeneration might be cell fusion. This process occurs in both a physiological and a pathophysiological context and can be affected by immune response due to inflammation. In this review the role of MSCs and cell fusion in tissue regeneration is discussed.

## 1. Introduction

Tissue regeneration is a physiological process that occurs during the whole life-span to maintain tissue homeostasis, but it also describes the ability to restore parts or even whole tissues or organs after injury or loss [1]. Additionally, this term is understood as a medical field in which this process is specifically induced and examined for trauma treatment. Organisms like the Mexican salamander axolotl [2,3,4,5] or the zebrafish [6,7,8] have a very high regenerative capacity, whereas this ability is very limited in humans [1]. Organs such as the liver, heart and pancreas and the central nervous system possess little regenerative capacity, while this ability is enhanced in the intestine, skin and hair. It is supposed that evolutionary “older” organisms possess a higher regeneration capacity than “younger” ones such as humans [9]. The same applies to development within a species. A fetus has higher potential for regeneration than an adult human. 

Clinical research focuses on agents, which facilitate tissue regeneration. Therefore, the process per se, but also all components involved in the regulation of this process have to be investigated. In short, important tasks that are performed after injury are the activation of the immune system, the removal of cellular debris, the induction of angiogenesis and the formation of new tissue [10]. Thereby, immune cells are one of the most important factors. Immune cells are recruited toward inflamed tissue, mainly regulating the healing process and therefore are involved in crosstalk with other cells involved in this process. One of these cell types are mesenchymal stem/stromal cells (MSCs), which are needed due to their ability to differentiate into cells of mesodermal lineage and their immunomodulatory activity [11,12]. In the clinic, MSCs and other stem cells, as well as different biomaterials are already used to enhance tissue regeneration [13]. Thereby, stem cells as well as their derivates have been applied. Extracellular vesicles are membrane vesicles of endocytic origin and are beneficial agents due to their small size, high reproducibility and low immunogenicity [14]. 

Another important aspect in tissue regeneration might be cell fusion, which, however, has been so far mainly investigated in developmental processes, such as myogenesis [15], osteogenesis [16] and placentation [17]. It is known that dysregulation of this process can lead to various diseases such as osteoporosis or preeclampsia [18,19] clearly showing the importance of cell fusion in these physiological processes. Nonetheless, several studies revealed that cells of bone marrow origin, such as hematopoietic stem cells and MSCs could restore degenerated tissue by adopting the phenotype of e.g., liver, neuronal, muscle and intestinal cells through cell fusion (for review see: [20,21]). Even though the necessity of cell fusion in tissue regeneration has been validated in several studies, the process itself is only scarcely understood, which particularly applies to factors and conditions that induce, mediate and terminate the merging of the plasma membranes of two (and more) cells (for review see: [18,22,23]).

An increasing body of evidence indicates the suitability of MSCs as promising cellular tools for tissue regeneration purposes, which is briefly discussed in this review.

## 2. The Role of MSCs in Tissue Regeneration

Stem cells are thought to be promising therapeutic agents due to their important role in the maintenance of tissue homeostasis and wound healing [24,25]. Thereby, MSCs are the most frequently used and best examined stem cells in the clinic. They are involved in bone regeneration, tissue repair and immune response and are distinguished by their anti-inflammatory properties and low immunogenicity [26,27,28]. In general, MSCs are multipotent cells, which are able to self-renew, but also to differentiate into cells of the mesodermal lineage. This characteristic can be investigated in vitro, where MSCs have to be able to undergo osteogenic, adipogenic and chondrogenic differentiation. This trait is beneficial in clinical use, because of the applicability of MSCs in diverse tissues. MSCs can be isolated from a wide range of tissues, such as adipose tissue, bone marrow and dental pulp, but also from peripheral, menstrual and umbilical cord blood [29]. Friedenstein was the first to describe MSCs, which he detected in bone marrow (BM) [30]. Thereby MSCs, isolated from the same tissue as well as from different sources, form a heterogeneous population, in which individual cells can vary in their phenotypes, differentiation capacities, proliferation rate and immunomodulatory potentials [29,31,32]. 

The role of MSCs in health and disease is as versatile as their appearance. On the one hand, MSCs have an anti-inflammatory effect and support the immune system and wound healing and on the other hand they have shown immune suppressing activity leading to a tumor promoting environment [33]. An important mechanism in tissue regeneration is the interaction of MSCs with the immune system [34]. The immune response in the microenvironment of inflamed tissue is provoked by the secretion of different molecules such as cytokines, chemokines and growth factors, which regulate the movement of MSCs via circulation towards the inflamed tissue—this process is named homing [35,36]. This homing property of MSCs can be used in the clinic, where MSCs can be some kind of transporter for therapeutic targets. Additionally, crosstalk between immune cells and MSCs is crucial for tissue repair [37,38,39,40,41]. MSCs are able to contribute to the healing process by secreting paracrine factors in turn. Beside MSCs as a whole, extracellular vesicles (EVs) are tested for therapeutic application. EVs are not only produced by MSCs, but by virtually all cells in the human body [42]. However, EVs isolated from different MSC sources can vary in their characteristics. Pomatto et al. demonstrated that EVs from the BM mainly promoted proliferation in a murine model of diabetic wound [43]. In contrast, EVs isolated from adipose tissue, mainly affected angiogenesis in the very murine model. Furthermore, EVs can also be altered to enhance therapeutic potential. Therefore, miRNA, siRNA or lncRNA can be loaded as cargo into the EVs. For example, EVs isolated from MSCs, which overexpressed lncRNA HOX transcript antisense RNA (HOTAIR), promoted angiogenesis and wound healing in diabetic mice [44]. In a study by Liu et al., it could be shown that the release of apoptotic bodies (a subgroup of EVs) by transplanted MSCs, led to the shift of macrophages towards an anti-inflammatory phenotype and promoted the healing of cutaneous wounds [45].

The properties of MSCs vary greatly depending on the tissue from which they are isolated, in which tissue they are used, whether in combination with other cells or scaffolds and whether the entire cell or just EVs are used. All of these variables make it difficult to predict the therapeutic success of MSCs in specific diseases more precisely. In the following, different disorders are described in which the use of MSCs and MSC-EVs showed a positive effect on the regeneration process.

## 3. MSCs and Their Derivates in Therapy

### 3.1. MSCs in Bone Diseases

Bone diseases such as osteoporosis, arthritis and periodontitis are a general problem in our population and the incidence of such diseases increases with age. These diseases often result from a malfunction of resorption and osteogenesis and inflammation even impairs bone destruction. Thereby, immune cells, inflammatory cytokines and MSCs play a crucial role in bone remodeling. Normally, the bone tissue of healthy people is able to continuously remodel itself throughout the whole life-span. If the self-healing process is disrupted, therapeutic measures have to be exerted. As the “gold standard”, the use of autogenous bone for transplantation is applied. Limitations include its availability and morbidity, which is why the use of MSCs and other natural or synthetic bone substitutes, as well as the combination of both has been further explored over time. One of those materials is termed SmartBone®, a biohybrid bone substitute [46]. Bari et al. used this scaffold and the lyosecretome, consisting of EVs and proteins, of MSCs to show that the lyosecretome improved bone formation [46]. Further synthetic bone grafting materials and xenografts in combination with MSCs were studied by Shiu et al. [47]. They used MSCs isolated from the BM and from the dental pulp and implanted them in combination with a synthetic material termed MBCP (micro–macro biphasic calcium phosphate) and Bio-Oss, a bovine-derived xenograft, and tested the effects on the healing process in a rabbit calvarial defect model. The combination of MSCs and grafting materials enhanced bone formation at the injury side, but it was not as effective as the application of autogenous bone. Thereby, they cultured the MSCs in 2D before the treatment, whereas studies of Kim et al. demonstrated that genes related to osteogenic processes were overexpressed in 3D culture systems [48]. Maybe the effect of MSCs and grafting materials would be greater if cells were cultured in 3D. 

There seem to be various factors being involved in the regulation of MSCs and their regenerative capacity. In addition to cultivation, the origin of the MSCs is another important factor, because the osteogenic capacity can vary between cells due to the usage of different signaling cascades [49]. Nevertheless, MSCs are the most interesting agents in bone remodeling. This includes the process of osteogenic differentiation, in which miRNAs within MSC-EVs seem to play an important role. This was investigated by Shirazi et al. [50]. They knocked down important regulators of the miRNA biosynthesis and observed a reduced differentiation rate of MSCs. 

Additionally, different paracrine factors can also have an impact on MSCs. It was demonstrated that the cytokine IL-1β, which is present at a relatively high concentration within the first 10 days after injury, inhibited the regenerative capacities of MSCs [51]. The innate immune system responses via interleukin-1 receptor, type 1 (IL-1R1)/MyD88 signaling, which in turn inhibits the Akt/GSK-3b/β-catenin pathway, resulted in a decreased proliferation, migration and differentiation capacity of MSCs. By the use of an IL-1R1/MyD88 inhibitor, the regenerative potential of MSCs could be improved. In cell culture studies, TGFβ, an anti-inflammatory cytokine, has inhibited the osteogenic differentiation of MSCs [49]. Similar has been reported for muscle cell differentiation [52]. All these studies demonstrate that the application of MSCs bears many benefits, however, there are several considerations to be taken into account, such as the origin of MSCs and additionally, interactions with the immune system might complicate the regeneration process.

### 3.2. MSCs in Muscle Diseases

Muscle regeneration, as well as bone regeneration, is a homeostatic process that regulates the healing of damaged tissue either through injuries or diseases affecting the muscles. Thereby, unipotent stem cells called satellite cells are the main component, which are attached to muscle fibers and stay in a quiescent state until they are activated due to damage signals [53]. MSCs do not possess myogenic differentiation capacity, but they are able to fuse to myoblasts to a small extent [54]. Differentiation capacity can be induced by the overexpression of Pax3 or β-catenin and by the satellite cell niche, but not by the niche’s factors IGF-1, IL-4, IL-6 or SDF-1 [55,56,57,58]. Additionally, MSCs showed a positive effect on muscle regeneration in Duchenne muscular dystrophy model mice by secreting the chemokine CXCL12 and osteopontin [59].

### 3.3. MSCs in Neurological Diseases

Multiple sclerosis (MS) is an autoimmune disease affecting the central nervous system (CNS) and is characterized by inflammation, demyelination and axonal degeneration [60]. Due to their immunomodulating and anti-inflammatory capacity, MSCs and their derivates are thought to be possible therapeutic agents for neurological disorders [61]. Various studies investigated the effect of MSCs or MSC derived EVs on MS in experimental autoimmune encephalomyelitis (EAE), an animal model of MS [60,62,63,64,65]. This includes Ahmadvand Koohsari et al., who demonstrated that the application of EVs from human umbilical cord blood MSCs reduced the amount of pro-inflammatory cytokines, such as IL-17a, TNF-α, and IFN-γ, leading to an alleviation of the disease [62]. Furthermore, the use of MSC-EVs, which have been stimulated by IFN-γ beforehand, enhanced motor skills and reduced neuroinflammation and demyelination, suggesting that stimulation of MSCs with pro-inflammatory cytokines might be necessary for an improved healing potential [63]. Adipose-derived EVs ameliorated EAE through effecting T-cell adhesion and proliferation, leading to a reduced demyelination and spinal cord inflammation [60,64]. In a different MS model, Theiler’s murine encephalomyelitis virus (TMEV) induced demyelinating disease, the administration of adipose-derived MSC-EVs showed similar results, resulting in attenuated motor skills and remyelination [66]. Additionally, MSC-EVs improved functional recovery in mice with a subcortical ischemic stroke, in rats after traumatic brain injury and in a rodent model of inflammation-induced brain injury [67,68,69].

### 3.4. MSCs in Cancer

Interestingly, MSCs are also thought to be suitable devices for cancer therapy. The benefit of MSCs is their homing capacity, which enables direct transport of the therapeutic target to the tumor tissue, which mimics an inflamed environment. Greco et al. demonstrated that MSC-EVs offer some benefits in comparison to normal cells, because they can be internalized by cancer cells to a greater percentage than normal cells [70]. Additionally, EVs are smaller and have been shown to be less immunogenic than MSCs, which is why they are able to carry chemotherapeutics such as paclitaxel [71] or doxorubicin [72] as well as anti-tumor RNA-based therapeutics such as different miRNAs [73,74,75,76,77,78]. However, MSCs might also possess a negative regulatory capacity in cancer treatment. Recent investigations are concentrating on the involvement of MSCs in the development of cancer stem/initiating cells (CS/ICs) either through the stimulation of secreted factors or by cell fusion [26,33,79]. MSCs have also been shown to either enhance or inhibit tumorigenicity [80,81,82,83,84]. The secretion of cytokines, MMPs and other molecules by MSCs could lead to modulations of the tumor microenvironment and to a switch of macrophages to a tumor promoting phenotype [33,85,86]. The phenotype of MSCs can thereby also be changed toward a more tumorigenic one due to the direct or indirect interaction with cancer cells. 

### 3.5. MSCs in Other Diseases

MSC and MSC-EVs have been effectively used in further diseases, some of the examples are discussed here. In liver fibrosis, IFN-γ pre-conditioned MSC-EVs induced anti-inflammatory macrophages and regulatory T-cells leading to tissue regeneration in a mouse model [87]. Similar has been demonstrated by Riazifar et al. in EAE [63]. In a rat urinary bladder augmentation model, MSCs and hematopoietic stem/progenitor cells were seeded onto different scaffolds and were transplanted into rats. This treatment promoted bladder tissue regeneration, partially through the formation of blood vessels [88]. The application of an ointment based on MSC’s secretome had an accelerating effect on skin wound healing in mice [89]. In contrast, the application of MSCs does not always have a direct effect on the diseases. In radiation-induced hematopoietic syndrome the administration of MSCs did not lead to recovery of the blood system in mice, but reduced lethality possibly due to a positive effect on other radiation-sensitive organs [90]. 

These results suggest that MSCs and MSC-EVs might ameliorate regeneration in different diseases through the regulation of immune cells, but how MSCs work and which factors regulate MSCs in turn has to be further investigated. A summary of the diseases described here in which MSCs or MSC-EVs, respectively have been used, can be found in Table 1.

## 4. MSCs and Cell Fusion in Therapy

### 4.1. Cell Fusion

Cell-cell fusion is a biological event, which plays a crucial role during embryonic development as well as in tissue regeneration or in muscle and bone formation [94,95,96,97]. This highly regulated process is not yet fully understood, but basically, the lipid bilayers of two different cells have to merge so that exchange of intracellular content is possible. Thereby, many factors regulate this process, starting with the genetically regulation, protein/fusogen expression and signaling cascades (Table 2). Due to high energetic and mechanistical barriers, which have to be overcome, spontaneous cell fusion is a rare process. Strong repulsive forces predominate between two cells, so that a morphological change, a bending of the two lipid bilayers, is necessary to overcome these forces [18,22,94,98]. Fusogens are proteins which are necessary for cell fusion. Well-known fusogens include, for example HAP2/GCS1 in plants [99,100], AFF-1 [101] and EFF-1 [102] in nematodes and syncytins in mammals [18]. Syncytins, a family of transmembrane proteins, are evolutionary relicts of a human endogenous retrovirus, which has been induced into the human genome during exogenous viral infections of germ cells, and regulate the formation of the placenta [94,103,104,105]. Therefore, numerous cytotrophoblasts fuse to form multinucleated syncytiotrophoblasts [104,105,106,107]. Additional factors aside from fusogens are known to promote cell fusion. These include EGF and TGFα [94,107]. Further studies have revealed that the fusogen syncytin-1 is also expressed in other cell types, such as osteoclasts, myoblasts and breast cancer cells, and might also be involved in the fusion of these cells [94,103,104,105]. Thus, these factors might play an important role in tissue regeneration.

### 4.2. Cell Fusion and Stem Cells

Further examples for physiological events in which cell fusion is necessary are the formation of a zygote, the development of skeletal muscles and the formation of multinucleated osteoclasts. The latter are important for bone resorption [112] and are generated by the fusion of macrophages. However, even though several factors have been identified that are involved in macrophage fusion, such as RANKL, DC-STAMP, MMP, E-Cadherin, CCL2, M-CSF, CD200 and CD47 [94,112], the process of macrophage fusion still remains unclear. Similar applies for myogenesis [94,110,113]. It is assumed that muscle progenitor cells might remain as satellite cells in their niche or differentiate into myoblasts, which in turn fuse to form primary multinucleated myofibers. The satellite cells are required in case of muscle growth and repair of muscle injuries, because myofibers have lost their proliferation capacity and are dependent on these cells. There are four major factors known regulating myogenesis—MYOG, MYOD, MYF6 (also termed MRF4) and MYF5—and there is evidence that upregulation of VEGFA and its receptors leads to an increase of cell fusion events [94,110,113]. MYMK and MYMX (or MINION) (Table 2) are additional fusogens that were recently investigated [108].

In conclusion, stem/progenitor cells seem to be the most fusogenic cell types beside macrophages and cells involved in developmental processes (such as trophoblasts and myoblasts) (Table 2). Not only in the early embryonic development, but also in post-natal tissues, stem/progenitor cells fuse with other stem/progenitor cells or differentiated cells to maintain tissue homeostasis including the growth and regeneration of tissues [111]. Especially the role in tissue regeneration is of interest for regenerative medicine, because mammals show a decreased regenerative capacity. The fusion of BMDCs has shown regenerative potential *in vivo*, e.g., in the CNS [114], in retinal tissue [115], in the liver [116] and in skeletal muscles [117,118].

Regenerative potential has been observed not only for stem cells of the BM, but also for stem cells of umbilical cord blood. Recently Collins et al. reported that they were able to fuse an immortalized human umbilical cord blood derived cell line (E12 MLPC) with normal human primary hepatocytes to produce a cell line with the expression profile and biological activity of mature hepatocytes, which can be cultured in vitro for a long time. Such cell lines are of importance for biological and clinical research as well as for personalized medicine [119]. It has been observed that the fusion between MSCs and cardiac cells and between MSCs and hepatocytes led to an ameliorated cardiac and liver, respectively, function [116,120]. In summary, the understanding of cell fusion processes and their involvement in many different physiological processes is essential for maintenance of a healthy status and might be important for the treatment of many diseases.

On the other hand, a dysregulation of this process could lead to severe diseases (Table 3). The overexpression of syncytins has been found in neurological diseases such as MS [105]. In contrast, during pregnancy a decreased expression of syncytins is correlated to preeclampsia, while defects in the fusion devices of oocyte and spermatozoid lead to infertility [105]. Osteoporosis and myopathy are linked to cell fusion defects of osteoclasts and myoblast [18]. However, not only defects, but also proper cell fusion events can lead to diseases. The best-known pathophysiological process involving cell fusion is the infection of host cells with enveloped viruses (Table 3). Viruses are dependent on host cells, which they can infiltrate and abuse for the replication of viral genetic information. Thereby, some of this viral genetic information became integrated into the human genome and interestingly normal human cells expressing retroviral envelope proteins have shown an increased fusogenecity [121,122,123]. 

A second disease in which cell fusion events might play a role is cancer (Table 3). Tumor tissue mimics a chronically inflamed environment, including signaling of apoptotic, hypoxic or inflamed conditions within the tissue and all of them promote cell fusion processes [109,124,125]. It is suggested that cell fusion might lead to the formation of CS/ICs as well as to tumor hybrid cells, expressing new genotypic and phenotypic characteristics. While the fusion in physiological processes leads to multinucleated cells, which have lost their ability to proliferate, it is suggested that fusion of cancer cells could result in hybrid cells with an enhanced proliferative capacity. Thereby, the development of chromosomal aberrations, like deletions, translocations and insertions—due to processes like heterokaryon to synkaryon transition and chromosomal missegregation—are characteristic for hybrids cells [126]. Additionally, cell fusion in cancer is related to increased tumorigenicity, aneuploidy, metastasis and therapy resistance [94,127]. Cell fusion in tumors—and thereby the formation of hybrid cells—leads to an enhanced heterogeneity including a high genetic and epigenetic variety [124]. 

An important factor involved in cell fusion induction might be TNFα. Its impact on cell fusion has been shown in breast cancer, breast epithelial and oral cancer, where it upregulates the expression of syncytin-1 [128]. Additionally, hypoxia, MMP9 and TNFα have been shown to be involved in induction of cell fusion of BC and breast epithelial cells via NFκB pathway [109,125]. By the use of the NFκB-inhibitor Minocyclin, cell fusion has been markedly reduced [129]. Additional cell fusion inhibitors have been used to inhibit HIV cell-free and cell-cell infections [130,131,132,133]. In summary, cell fusion is an important process in tissue regeneration, but it also might lead to the development of different diseases, if this process is dysregulated.

## 5. Conclusions

In summary, MSCs are beneficial therapeutic agents due to their stem cell characteristics, immunomodulatory potential, low immunogenicity and homing capacity. They can regulate tissue regeneration by paracrine signaling and/or direct differentiation [28]. EVs, isolated from MSCs, show similar therapeutical characteristics as MSCs, but due to their small size and even lower immunogenicity they seem even more suitable. In animal models the application of MSCs and MSC-EVs, has been tested in various diseases such as osteoporosis, MS and Duchenne muscular dystrophy [59,61]. A recent search at *clinicaltrials.gov* with the key term “mesenchymal stem cell” yielded in over 300 completed clinical trials, where MSCs have been used in a huge range of diseases such as Parkinson’s disease, MS, type 2 diabetes, liver failure and many more. The search for therapeutic agents increases and hence, the examination of MSC applicability. Thereby, the conflictive characteristics of MSCs must be considered as well as the possibility that MSC not only are involved in tissue regeneration, but might also promote tumor growth by modulating the inflamed microenvironment or through cell fusion. Another important factor is the multitude of external factors that influence the effectiveness of MSCs and MSC-EVs. This includes the origin of the cells, previous stimulations, culture conditions and many more.

Additionally, the role of cell fusion in tissue regeneration and in therapy must attract more interest in research. Especially, because it is already known that dysregulation of this process can lead to different diseases such as MS or preeclampsia [105]. Thereby, also the role of fusogens has to be examined, because these factors might be useful as therapeutic agents as well. In the end, there are still many open questions, but also new approaches to improve therapeutic effectiveness.

## Figures and Tables

**Table 1:** Overview of diseases in which MSCs or MSC-EVs have been applied.

Disease	MSC or EV	Reference
Bone disorders	MSC	[46,47,49]
	EV	[50]
Duchenne muscular dystrophy	MSC	[59]
MS	EV	[60,62,64,66]
Subcortical ischemic stroke	EV	[67,91]
Brain injury	EV	[68,69,92]
Liver fibrosis	EV	[87]
Urinary bladder augmentation	MSCs	[88]
Skin injury	EV	[89]
Graft-versus-Host-Disease	EV	[93]

**Table 2:** Overview of factors involved in cell fusion.

Fusion-Promoting Factors		Source	Reference
Fusogens:	Syncytins (mammals)	Placenta, breast cancer, osteogenesis	[18]
	HAP2/GCS1 (plants)	Gamete fusion	[99,100]
	AFF-1 and EFF-1 (nematodes)	Epidermal, vulval and pharyngeal fusion events	[101,102]
MYMK and MYMX		Myogenesis	[108]
TNFα		Cancer	[109]
EGF		Syncytialization	[107]
VEGFA		Myogenesis	[110]
Stem cells		(Mainly) tissue homeostasis	[111]
Macrophages		Osteogenesis	[112]
Trophoblasts		Placenta	[107]
Myoblasts		Myogenesis	[113]

**Table 3:** Examples of diseases in which cell fusion or fusogens, respectively, is impaired.

Disease	Reason	Referenece
neuronal diseases	overexpression of syncytins	[105]
preeclampsia	decreased expression of syncytins	[19]
infertility	defects in the fusion of sperm and egg	[105]
viral infections	virus × host cell fusion	[123]
cancer	cancer cell × cancer or normal cell fusion	[81]
osteoporosis	defects in macrophage fusion	[18]
myopathy	defects in myoblast fusion	[18]

## Abbreviations

BM	Bone marrow
CNS	Central nervous system
CS/IC	Cancer stem/initiating cell
EAE	Experimental autoimmune encephalomyelitis
EV	Extracellular vesicle
HOTAIR	lncRNA HOX transcript antisense RNA
IL-1R1	Interleukin-1 receptor, type 1
MBCP	Micro–macro biphasic calcium phosphate
MS	Multiple sclerosis
MSC	Mesenchymal stem/stromal cell
TMEV	Theiler’s murine encephalomyelitis virus
